# Differential effects of human density, environmental health, and group size on urban coyote detection, boldness, and exploration

**DOI:** 10.1038/s41598-025-21946-y

**Published:** 2025-10-30

**Authors:** Cesar O. Estien, Lauren A. Stanton, Christopher J. Schell

**Affiliations:** Department of Environmental Science, Policy, and Management, University of California– Berkeley, Berkeley, CA 94720 USA

**Keywords:** *Canis latrans*, Risk-taking, Human-wildlife interactions, Novel object, Urbanization, Carnivore behavior, Pollution, Animal behaviour, Urban ecology, Behavioural ecology

## Abstract

Comparative studies show that urban coyotes behave differently from rural counterparts. However, these studies often homogenize cities. Cities feature diverse pressures for wildlife, such as variation in human densities and environmental health, two factors known to increase risk-taking. This heterogeneity creates a landscape of risk, which may drive locally adapted behavioral strategies within cities. Yet, the influence of these pressures on coyote behavior remains unclear. To investigate this, we conducted novel object testing at 24 sites across gradients of human density and pollution. We recorded coyote detections, group size, and behavioral responses to the novel object, focusing on time spent alert, time spent close, and total exploration. We found that coyote detections varied with human density and pollution, with markedly lower detections in areas with high human density and pollution. Coyote boldness (time spent alert and close) and exploration were uniformly associated with human density, with coyotes in high human density areas displaying elevated boldness and heightened exploration. We also found that time spent close and exploration increase with group size. In contrast, coyote risk-taking did not vary with pollution. Our results suggest that heterogeneity in human density, environmental health, and social context differentially affects coyote ecology, which may have consequences for human-carnivore coexistence.

**Supplementary Information:**

The online version contains supplementary material available at 10.1038/s41598-025-21946-y.

## Introduction

Cities are complex, coupled human-nature systems that establish variable landscapes of risk that pressure wildlife to rapidly adjust or face local extirpation^1–3^. Because urban ecosystems challenge organisms with novel scenarios and disturbances that encourage phenotypic differentiation, research to date has focused on understanding how urban and rural populations differ. In particular, understanding how urban and rural individuals contrast in behavior, including boldness—an individual’s response to a risky situation or event—and exploration—an individual’s willingness to investigate a novel object or situation—has been of large interest. This is because behavior is a particularly flexible trait that organisms alter in response to environmental cues. Indeed, urban individuals have been shown to diverge from their rural counterparts in several behaviors, including boldness, exploration, and aggression^3,4^. Yet, this research approach has often treated cities as homogeneous, despite the strong landscape heterogeneity within and across cities^5,6^. Within-city variation in disturbances, including humans and pollutants, may therefore also drive phenotypic divergence at a fine scale, perhaps even across neighborhoods^7^.

Human presence is typically cited as one of the most prominent factors driving alterations in wildlife ecology, including behavior^8^. For instance, urban wildlife are less likely to flee from humans compared to their rural counterparts^9–11^, and this heightened tolerance is often used as a metric of boldness^12,13^. However, anthropogenic pressures (e.g., population density, foot traffic, building density) vary across neighborhoods and can have differential effects on wildlife behavior, with larger carnivores, such as mountain lions (*Puma concolor*) and bobcats (*Lynx rufus*), reducing activity in, or avoiding, areas with high human presence^14,15^. Indeed, humans can mediate trophic interactions and shape predator assemblages across the urban matrix, allowing prey and smaller predators to avoid predation by selecting habitats with higher human densities or development^14,16,17^; this phenomenon is called the human shield hypothesis^18^. However, organisms in areas with high human densities face trade-offs: their access to natural habitat is relatively limited, with increased exposure to anthropogenic disturbances. Relaxed predation pressures and reduced natural habitat may therefore combine to bolster boldness and exploration in human-dominated environments^19,20^, leading to the exploitation of anthropogenic resources for food and shelter. Variation in these pressures, as well as species-specific responses to them, should induce behavioral variance across neighborhoods and species in a single city^7^.

In addition to the amount of natural habitat, environmental health can also influence animal behavior. Environmental health, often measured by the concentration of pollutants (e.g., ozone, pesticides, heavy metals)^21^, can serve as a proxy for habitat quality and degradation, varying spatially due to legacies of injustice. For instance, historical redlining, a policy that denied credit and financial services to individuals based on ethno-racial identity^22,23^, has been linked to reduced environmental health (i.e., more pollutants) across the United States along with, for example, reduced vegetation, low tree canopy, elevated temperatures, and higher noise levels relative to non-redlined neighborhoods^24–28^, with downstream consequences on wildlife biodiversity^29,30^. This subsequent variation in environmental health may impact wildlife risk-taking through two main pathways. First, most human-produced pollutants are endocrine disruptors, altering the hormonal systems that are known to underpin behavioral traits like boldness and exploration^31–34^. Birds exposed to metal pollution were slower explorers, had altered migratory behavior, and reduced song repertoires^35–38^, while fish exposed to pollutants were generally bolder and exhibited slower or decreased exploration tendencies^39^. Thus, pollutants can alter the behavioral traits that are critical in establishing and succeeding within cities, including boldness and exploration^31,40–42^. Second, lower environmental health corresponds to a reduction in habitat quality (e.g., less canopy, high impervious surfaces, reduced vegetation^26,28^) and often less diverse species pools^30,43^, which may lead to altered behavior due to a reduction in predation pressure and interspecific competition^17,44–47^. Lastly, because resources are limited in areas with poorer environmental health (e.g., less green spaces, less prey diversity), carnivores may be more likely to occur in neighborhoods with better environmental health^43^. Despite these potential pathways, variation in environmental health within cities has not yet been fully integrated into urban carnivore ecology (but see Wilkinson et al.^48^ and Hentati et al.^43^).

Urban mesocarnivores, such as coyotes (*Canis latrans*), are an ideal model for investigating urban-induced behavioral changes^49^. Urban coyotes, an apex predator in cities and flagship species for human-carnivore conflict, are bolder and more exploratory than non-urban coyotes^50,51^. Behavioral adjustments, such as diet flexibility^52^ and movement strategies^53^, have greatly facilitated their adaptation to urban contexts across biomes and human disturbance regimes^49^; however, these same shifts have exacerbated human-carnivore conflict^54–56^. Though myriad studies have examined how coyotes are adapting to cities behaviorally along axes of urbanization, the effect of environmental health on coyote risk-taking remains uninvestigated. This is particularly pressing as disruptions to their behavior can alter community dynamics and ecosystem function by disrupting the top-down pressures coyotes exert on other wildlife. Disentangling how human disturbances shape carnivore behavior is critical to maintaining healthy, functioning ecosystems.

To address these gaps, we used novel object testing to elicit a behavioral response^50,57,58^ and explore how human density and environmental health were associated with coyote risk-taking behaviors (i.e., boldness and exploration). We also explored how the number of coyote detections varied with our landscape variables. First, we predicted that coyote detections would be lowest in sites with high human density and high pollution areas as a result of reduced habitat availability and quality^43^. Second, we hypothesized that coyote risk-taking would vary with human density and pollution. Specifically, we predicted that high human densities would be associated with elevated boldness and exploration as previous work has demonstrated that carnivores in high human density environments show increased trait values for both behaviors^50,59–62^. We also predicted that areas with high pollution (and thus, more anthropogenic disturbances) would be associated with elevated boldness and increased exploration.

## Methods

### Study area

**Fig. 1:**
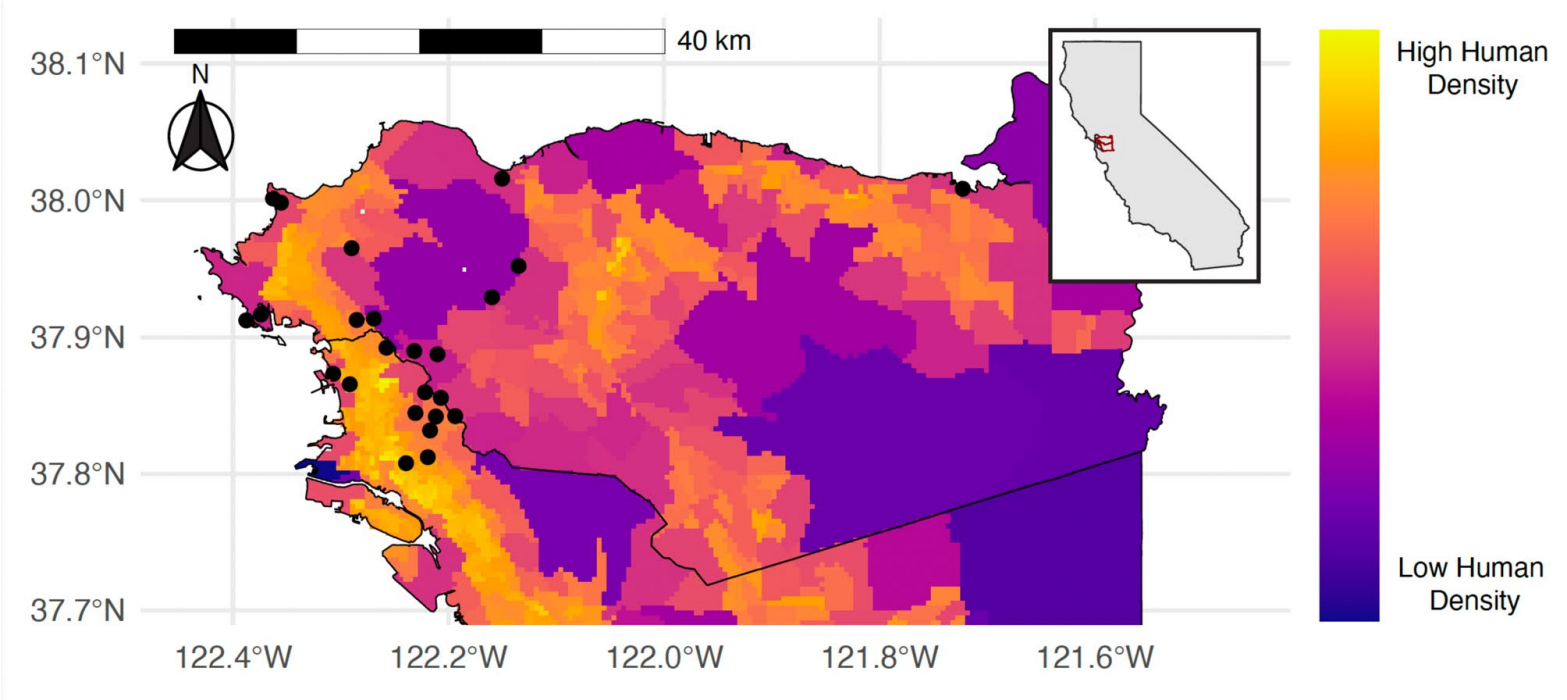
Study Area Overview. A map of the experimental sites located in the East Bay Region of the Bay Area in Northern California across human population density. Each dot represents a camera. This map was generated using R version 4.4.2 (https://www.r-project.org/).

This research was conducted at 15 public parks and 9 private residences across the East Bay region of Northern California between December 2022 and February 2023. The study included sites within the cities of Antioch (2022 estimated population density: 3,906 people per square mile), Berkeley (12,401 ppl/mi^2^), Oakland (7,725 ppl/mi^2^), and Richmond (3,758 ppl/mi^2^)^63^ (Fig. 1). The East Bay features a Mediterranean climate, characterized by mild temperatures throughout the year and wet, cooler winters—the conditions during the study period (mid-winter average high of 13 °C and low of 6 °C)^64^. Motion-activated trail cameras (Bushnell, Overland Park, Kansas, USA) were used at each location and operated for an average of 66 days (range: 45–84 days). Each camera recorded 30-s clips with a one-second interval between triggers. Cameras were deployed only at locations where landowner consent was secured, with all sites spaced at least 1 km apart to align with territorial ranges typical of urban mammals, as established in previous studies^65–67^.

### Geospatial processing and data analysis

We created a buffer around each camera site at 500 m for extracting our landscape variables of human population density (people per square mile)^68^ and pollution^69^. Due to a minimum camera spacing of 1 km, we placed a 500 m buffer to avoid potential spatial pseudo-replication between sites (similar to Lombardi et al.^70^, for example). For pollution, we used similar methods to Estien et al.^26^ and downloaded environmental hazard variables from CalEnviroScreen^69^ to create a pollution burden score for each site. We extracted the mean for the 10 variables: particulate matter_2.5_ (PM_2.5_; particles in the air with a diameter of 2.5 µm); diesel PM_2.5_ (PM_2.5_ emitted by diesel engines), toxic air contaminants (harmful chemicals released by industrial facilities), cleanup sites (i.e., brownfield sites), groundwater threat (the type and amount of leaking underground storage tanks as well as sites that pose a risk for contaminating underground water), hazardous sites (sites that generate, treat, and dispose of hazardous waste), solid waste sites (presence of landfills, garbage transfer stations, composting, and recycling sites), lead risk from housing (percentage of houses with likelihood of lead-based paint), traffic, and ozone. We then averaged these variables and created a percentile such that a score of 0 would represent no pollution burden and 100 would represent a high pollution burden^26^ (see Estien et al.^26^ for more detailed methods). After extracting the mean for human population density and pollution per site buffer (Figure S1), we calculated the median in our dataset and subsequently categorized each site as ‘low’ or ‘high’. All geospatial analyses were conducted in ArcGIS Pro using the ‘Zonal Statistics’ tool to extract the mean for all hazards.

Prior to selecting human population density and pollution, we considered myriad landscape variables related to anthropogenic disturbances and habitat quality – specifically median household income, artificial light at night, vegetation via normalized difference vegetation index (NDVI), percentage of impervious surfaces, housing density, and road density. These variables were highly correlated with our pollution burden metric and/or human population density (Figure S2); therefore, we excluded these variables from our final models. We retained pollution burden, rather than NDVI, in our analysis because it offers a more comprehensive measure of habitat quality. Although NDVI is often used as a component of habitat quality, it is highly correlated with several anthropogenic variables, including population density, road density, impervious surface, and light pollution. In contrast, our pollution burden metric incorporates direct measures of environmental contaminants, such as air pollutants, hazardous waste sites, and lead exposure risk and is less associated with other anthropogenic variables while being strongly correlated with NDVI, indicating that it captures a distinct dimension of overall habitat quality.

### Novel object test and behavioral coding

**Fig. 2:**
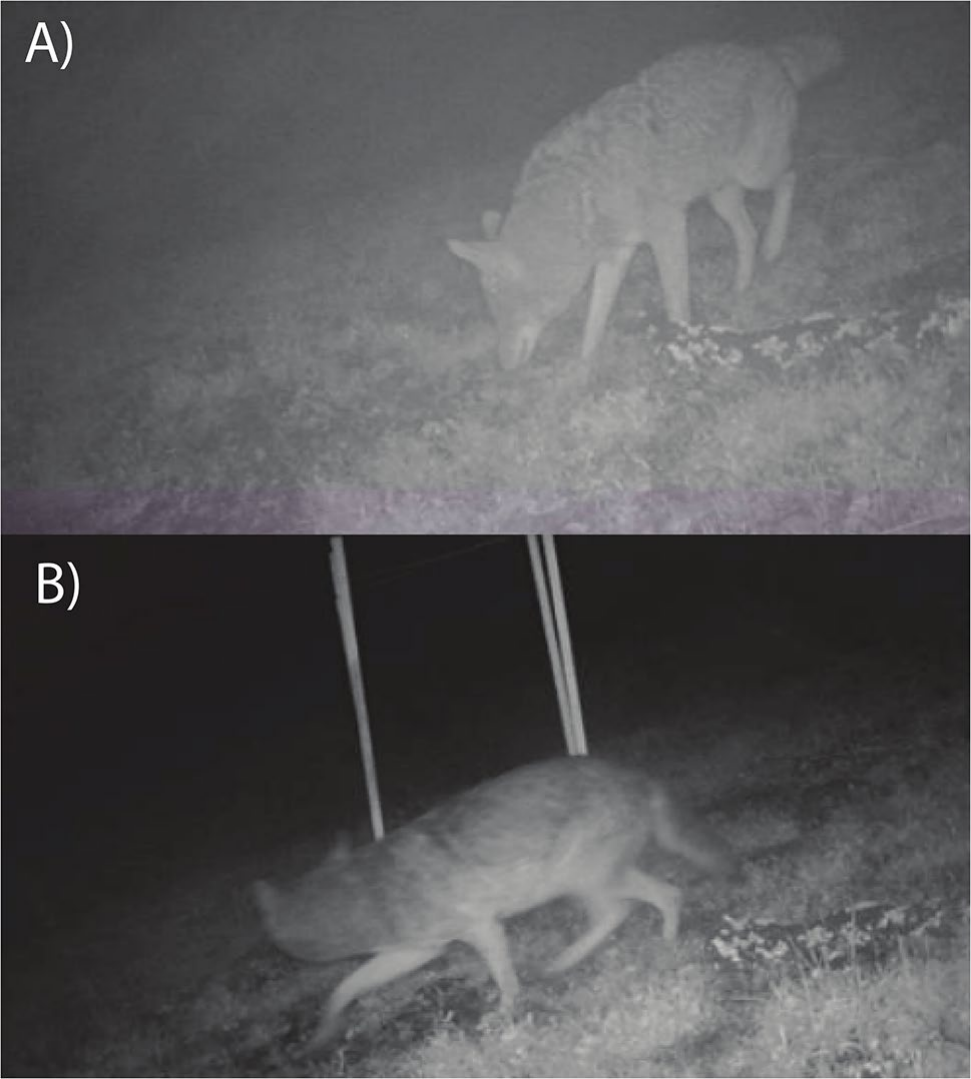
Images of coyotes interacting with our treatments. The top image (**A**) shows a coyote interacting during the control period (attractant only). The bottom image (**B**) shows a coyote interacting during the novel object period (attractant and novel object). Photo credit: Cesar Estien.

We used a paired-site design where each site (*n* = 24) served as both a control and treatment, with the order of condition randomized across sites. At each site, the testing period lasted at least 8 weeks and followed the methods in Breck et al.^50^. Briefly, we dug a shallow hole in the ground and then filled it with a tablespoon of bait (Sweet Meat Predator Bait, Russ Carman, New Milford, Pennsylvania). The hole was then covered with the removed substrate. We placed a fatty acid scent tab (Pocatello Supply Depot, Idaho) on top of the covered hole as an additional attractant^50^. Instances where only the bait and fatty acid tab were applied served as our control (Fig. 2A). For our treatment, we added a novel object consisting of four wood stakes in a 1m^2^ square formation around the hole, with a single white rope threaded across the top of all stakes standing roughly 1 m above the ground (Fig. 2B)^50^. For the first four weeks, a site was randomly given the control (only attractant) or the treatment (attractant + novel object). For the last four weeks, if the site had first received the control, it then received the treatment, and vice versa. Between each testing condition, we replenished the attractant. For each site, we coded several behaviors of interest (Table S1) and recorded the number of detections per species. For detections, videos within 30 min of each other were removed to ensure the independence of species triggers (i.e., temporal independence)^43,71^.

To understand coyote responses to our novel object, we extracted data from each video (i.e., an observation) using Behavior Observation Research Interactive Software (BORIS)^72^. Any video that fell within 30 s of the previous video was considered the same observation with the same individuals, as we did not consistently observe coyotes lingering around longer than that (average time between behavioral observations = 360 min). We recorded several behaviors, listed below and in our ethogram (see Table S1), based on Breck et al^50^. However, our ethogram slightly deviates from Breck et al. ^50^ in several ways. First, we focused on general ‘alert’ behavior (i.e., being attentive to the surrounding environment) rather than solely vigilance to better capture an individual’s perception of risk in their surrounding landscape. Second, we focus on broad exploratory behaviors to capture several exploration-related behaviors, including digging, which was categorized as a ‘comfortable’ behavior in Breck et al^50^. Next, we did not include comfort behaviors (i.e., urination, rub-roll, defecation, consumption of attractant) in our analysis as they made up a small portion of our behavioral occurrence data, leading to model convergence issues. Lastly, we created a single ‘time spent close’ metric (i.e., being within one body length of the novel object), instead of dividing proximity-related behaviors into time spent far, close, and on the attractant to reduce observer errors in accurately recording these spatial behaviors.

Within an observation, we recorded time spent alert regardless of an individual’s distance to the object. We recorded our remaining behaviors within one body length of the object. When a species was within one body length of the object, we recorded the amount of time it spent close. Time spent close also included time spent within the object’s interior (i.e., between the wood stakes). We consider time spent close and time spent alert as our metrics of boldness. We then measured coyote exploration by recording the number of occurrences of the following behaviors: digging, sniffing, touching, and moving through the object. We then calculated the total number of times an exploratory behavior occurred for each individual (i.e., total exploration). Thus, we identified bolder animals as ones that spent less time alert and more time close, and more exploratory animals as ones that had a higher total exploration metric.

Prior to coding, four observers were trained on the same 35 videos that we chose at random until > 80% interobserver reliability was achieved. We used Cohen’s kappa to assess interobserver reliability^73^, which was 85.67%. After coding was complete, behavioral data were cleaned to ensure the absence of behaviors (e.g., a coyote was not observed to be alert) was also captured (Supplemental Materials 1). For observations with multiple coyotes, we summarized behaviors into a single data point per observation to avoid pseudo-replication. To preserve the extremes of risk-taking behavior, we extracted the most risky value rather than an average: the minimum time spent alert, the maximum time spent close, and the maximum amount of exploration per observation.

### Data analysis

We conducted a preliminary analysis for our behavioral data via linear mixed models with a Gaussian distribution in the *lmerTest* package^74^. We examined the following variables in our preliminary analysis to understand which variables to retain in our final analysis: testing condition order (i.e., control then treatment, or treatment then control), testing condition (control or treatment), total coyote detections, group size, and observation number. We found that testing condition order and total coyote detections per site did not significantly affect the behaviors we coded for. Both observation number (i.e., the chronological order of observations made at a site per treatment) and testing condition had a significant negative effect on each coyote behavior, while group size significantly positively influenced time spent close and total exploration. Lastly, we found that our data was zero-inflated. Thus, we included observation number as a fixed effect across our final models to control for the negative effect of time, which we expect was due to loss of potency, or complete removal, of the attractant over time (e.g., dissipation, consumption, moving of the attractant by a squirrel), and retained group size and treatment as variables of interest. We did not find group size to be correlated with population density (Pearson’s correlation: 0.050) or pollution burden (Pearson’s correlation: -0.110).

For our detection models, we similarly explored potential influential variables, focusing on testing condition order and the number of days the camera was active, to refine our variables. We did not find a significant influence of testing condition order (Welch’s t-test *p* = 0.884), nor number of days active (Pearson’s correlation: 0.119; Figure S3) on the total number of detections observed at a site. To determine if human density and pollution had a significant effect on the total number of coyote detections at a site, we used a negative binomial model in the *glmmTMB* package^75^. Human density and pollution were fixed effects, site was a random effect, and we included the number of days the camera was active per site as an offset variable, following similar methods to Hentati et al.^43^.

To test whether human density and pollution burden were associated with the observed behaviors, we used a zero-inflated negative binomial mixed models in the *glmmTMB* package to account for the high number of zeros in our dataset^75^. We created 11 candidate models to assess the suitability of various combinations of fixed effects, including a null model and global model (Table S2). Our null model had observation number as the only fixed effect. Site was included as a random effect across all models. Model fit was assessed using Akaike’s information criterion (AIC) corrected for small sample size^76^. Models within two ΔAICc were considered to be equally likely as our best-performing model. From the best-performing model, we extracted estimates (β), p-values, confidence intervals (CIs), and R-squared goodness of fit values (R^2^). To compare the behavioral responses between variables in our models, we conducted Tukey’s pairwise comparisons using estimated marginal means from the best-performing model. Lastly, we examined correlations between behaviors using Spearman’s correlation.

## Results

### Detections

Across 24 sites, we received 274 coyote detections in total across 14 sites (Table 1). Human densities negatively affected coyote detections (ꞵ = -1.625, CI: -3.056, -0.194, p < 0.05; Figure S4-5; Table S3), with 48 detections in high human density areas compared to 226 detections in low human density areas. Similarly, pollution negatively affected coyote detections (ꞵ = -3.031, CI: -4.991, -1.071, p < 0.01; Figure S4-5; Table S3), with 36 and 238 detections in sites with high and low pollution burden, respectively.

**Table 1:** Coyote detections in relation to human density and pollution.

Variable	Number of sites	Number of sites visited by coyotes	Total visits by coyotes
Low human population density	12	8	226
High human population density	12	6	48
Low pollution	13	11	238
High pollution	11	3	36

### Boldness and exploration

In total, we recorded 279 behavioral observations for coyotes with 171 during the control and 108 while the novel object was present. We found that vigilance was not strongly correlated with time spent close (Spearman’s correlation = 0.42) or total exploration (Spearman’s correlation = 0.41), while time spent close and total exploration were strongly correlated (Spearman’s correlation = 0.83) (Figure S6). These relationships remained true when we disaggregated the data into high and low human density, as well as high and low pollution burden.

**Fig. 3:**
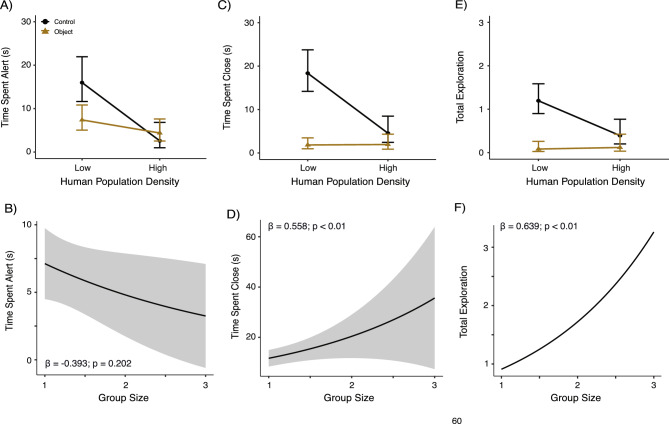
Coyote risk-taking in relation to human density and group size. Panels show (**A-B**) time spent alert, (**C-D**) time spent close, (**E–F**) total exploration in relation to human population density categories (top row) and group size (bottom). The control (attractant) is shown in black and marked with a circle, and the treatment (novel object and attractant) is shown in brown with a triangle. Shaded areas represent 95% confidence intervals.

Our top model for time spent alert was our human density-treatment interaction model and held most of the support (weight = 0.72), indicating that human density and our treatment interacted to affect the amount of time coyotes spend alert (Table 2; S2). Generally, coyotes decreased their time spent alert in areas with high human density, and while the novel object was deployed (Table 2). When considering the interaction between human density and treatment, we had three findings. First, during the control period (i.e., attractant only), we found that coyotes spent significantly more time alert at sites with low human density (Tukey’s ꞵ = 1.819, p < 0.01; Fig. 3A). Second, we during the object period (i.e., novel object and attractant), we found no significant differences between sites with low and high human density (Fig. 3A). Lastly, when we compared behavioral responses to each treatment per human density category, we found that coyotes at sites with low human density were significantly less alert during the object period relative to the control (Tukey’s ꞵ = -0.771, p < 0.01), whereas coyotes at high human density sites showed no significant differences (Fig. 3A). We found no effect of pollution burden (Table S2) or group size (Table S2; Fig. 3B) on time spent alert.

**Table 2:** Parameter estimates for best-performing coyote risk-taking from zero-inflated negative binomial mixed models. Significant terms are bolded.

Behavior	Model	R^2^_c_	Term	Estimate	SE	Pr ( >|z|)	95% CI
Time Spent Alert	Human Population Density * Treatment	0.380	**Intercept**	**2.096**	**0.146**	** < 0.001**	**1.282, 2.911**
			**Human Density (High)**	**1.399**	**0.433**	** < 0.01**	**0.550, 2.248**
			Treatment (Object)	0.036	0.476	0.940	-0.897, 0.969
			**Observation Number**	**-0.049**	**0.011**	** < 0.001**	**-0.069, -0.028**
			Human Density (High) * Treatment (Object)	-0.806	0.527	0.126	-1.839, 0.227
Time Spent Close	Global	0.526	**Intercept**	**3.013**	**0.316**	** < 0.001**	**2.395, 3.634**
			Pollution Burden (High)	0.255	0.272	0.349	-0.279, 0.789
			**Treatment (Object)**	**-1.793**	**0.264**	** < 0.001**	**-2.310, -1.277**
			**Human Density (High)**	**-1.395**	**0.319**	** < 0.001**	**-2.019, -0.770**
			**Group Size**	**0.558**	**0.214**	** < 0.01**	**0.139, 0.976**
			**Observation Number**	**-0.063**	**0.009**	** < 0.001**	**-0.080, -0.046**
			Pollution Burden (High) * Treatment (Object)	-1.008	0.655	0.124	-2.291, 0.275
			Human Density (High) * Treatment (Object)	**1.448**	**0.470**	** < 0.01**	**0.526, 2.370**
Total Exploration	Global	0.601	Intercept	0.460	0.306	0.132	-0.139, 1.059
			Pollution Burden (High)	0.371	0.297	0.213	-0.282, 0.954
			**Treatment (Object)**	**-2.118**	**0.358**	** < 0.001**	**-2.820, -1.412**
			**Human Density (High)**	**-1.114**	**0.335**	** < 0.001**	**-1.772, -4.571**
			**Group Size**	**0.639**	**0.200**	** < 0.01**	**0.237, 1.030**
			**Observation Number**	**-0.088**	**0.014**	** < 0.001**	**-0.114, -0.061**
			Pollution Burden (High) * Treatment (Object)	-1.111	1.147	0.333	-3.360, 1.138
			Human Density (High) * Treatment (Object)	**1.477**	**0.580**	** < 0.05**	**0.340, 2.614**

The top model for time spent close was our global model (weight = 0.87), followed by our human density-treatment model (weight = 0.12), providing strong support for the effect of human density and treatment on this behavior (Fig. 3C; Table 2; S2). We found similar trends for time spent close observed in time spent alert, with coyotes generally spending less time close while the novel object was present and in areas with high human density (Table 2). We also found that as the number of individuals within an observation increased, coyotes spent more time close overall (Fig. 3D; Table 2). During the control period, we found that coyotes spent significantly more time close to the attractant at sites with low human density (Tukey’s ꞵ = 1.395, p = 0.001; Fig. 3C). In contrast, during the object period, we found no significant differences between sites with low and high human density (Fig. 3C). When we compared behavioral responses to each treatment per human density category, we found that coyotes at sites with low human density were significantly less close during the object period relative to the control (Tukey’s ꞵ = -2.297, p < 0.001), whereas coyotes at high human density sites showed no significant differences (Fig. 3C). We did not find an effect of pollution burden on time spent close (Table 2).

Lastly, for total exploration, the top model was our global model (weight = 0.81), followed by our group-size treatment model (weight = 0.13), indicating that group size alongside landscape variables strongly affect coyote exploration (Figs. 3E-F; Table 2; S2) We found similar trends in exploration as seen in time spent alert and close, with coyotes exploring less while the novel object was deployed and at sites with high human density (Table 2). Similar to time spent close, we found that as the number of individuals within an observation increased, coyotes expressed more exploratory behaviors (Fig. 3F). When we compared exploration during the control period, we found that coyotes explored significantly more at sites with low human density (Fig. 3E). We did not find any differences in coyote exploration when we compared the object period. Finally, when we compared the control and novel object period, we found that coyotes at sites with low human density explored significantly less during the object period relative to the control (Tukey’s ꞵ = -2.306, p < 0.001), whereas coyotes at high human density sites showed no significant differences (Fig. 3E). We did not detect an effect of pollution burden on total exploration (Table 2).

## Discussion

Here, we provide evidence suggesting that within-city variation in human densities and environmental health affect coyote ecology. First, we found that the number of coyote detections varied with both human density and pollution, with fewer coyote detections in areas with high human density and high pollution. We also found that pollution had a stronger negative effect on coyote detections than human density, suggesting that environmental health may be a better predictor of coyote activity than human density. Second, treatment-related differences in coyote boldness and exploration were absent in high-density human environments. Lastly, we found that group size influenced two key risk-taking behaviors alongside human density: time spent close and total exploration. These results suggest that group size and factors associated with human density, such as risk and habituation, drive coyote risk-taking rather than environmental health. Overall, our results suggest that human densities, environmental health, and group size differently affect aspects of coyote ecology, offering insight into how coyotes are adapting to urban environments.

Consistent with recent research^43^, we found that coyote detections were markedly lower in areas with higher human densities and higher pollution. This is likely because although coyotes are highly adapted to cities, both societal and ecological factors can limit their establishment across the urban matrix. Coyotes inhabiting high human density areas can reap the high rewards of anthropogenic food or other resources (e.g., unclaimed territories); however, they also face tension with humans^77,78^, resulting in conflict^55,56^ and removal^79^. Futhermore, high human density areas can create finite prey resources due to a high concentration of buildings and other impervious surfaces, priming situations for potential negative human-coyote interactions to occur – such as raiding trash cans, moving through yards for food, or extremes like attacking pets. In parallel, ecological resources similarly limit coyotes in urban areas. Within our study area, ecological resources that coyotes rely on to successfully establish a territory, such as green space availability, are not evenly distributed as a result of societal inequities^80^. Lastly, areas with high human densities and pollution tend to have a higher concentration of infrastructural barriers, such as the 580 highway, that may impede the dispersal and movement of coyotes from greenspaces in richer habitats into areas with higher pollution. Thus, coyotes in our study area are detected less in more polluted areas, likely due to limited green space, reduced vegetation, and movement barriers.

We found strong support for our human density hypothesis in coyotes, supporting previous literature showing behavioral variation in coyotes as a consequence of urbanization^50,51,59,61,62^. First, at high human density sites, coyote alertness and proximity did not differ between treatments, whereas at sites with low human density, coyotes spent less time close and alert while the novel object was present. Second, in the control condition, coyotes inhabiting low human density areas spent more time alert and close to the attractant, suggesting heightened wariness of human presence. Coyotes are usually wary to novel stimuli in both field and captive settings^81–83^. However, individuals with greater experiences of people in both the wild^59^ and captivity^84^ display greater tolerance of human presence and infrastructure. In addition, increased consumption of human food subsidies in cities is thought to promote coyote boldness via associative learning^85,86^, which may reduce fear of humans and associated disturbances (e.g., objects, sounds)^59,84,87,88^. Additionally, the absence of “traditional” apex predators (e.g., mountain lions and wolves) that might otherwise temper risky behaviors could also encourage coyote boldness in cities^19,89^. Consistent treatment-related responses in high human density areas further support the assertion that increased human densities promote coyote tolerance to human-induced novelty.

We also found that coyote exploration, as measured by total exploratory behaviors, is driven by human density. Urban coyotes are known to be more exploratory than their rural counterparts^86^. This behavior likely occurs due to urban coyotes having a higher frequency of encountering novelty, creating an extensive history of exploring novel features that can lead to habituation.

For instance, coyotes in areas with high human densities may be exposed more frequently to stimuli such as trash cans, scents, and human infrastructure, compared to coyotes in areas of lower human density. Our finding that coyotes at sites with high human density do not significantly change the number of exploratory behaviors directed towards the novel object, unlike coyotes at sites with low human density which reduced their exploration, further supports this hypothesis. However, further research is needed to explicitly investigate if coyotes increase or decrease their exploration when exposed to novel stimuli repeatedly (but see Garcia, Parsons, and Young^90^ and Young, Touzot, and Brummer^91^).

Both time spent close and exploration were found to be strongly correlated with each other, indicating that an individual’s tendency to take a risk by approaching a novel object is tightly coupled with their willingness to also explore. Additionally, we found that both time spent close and total exploration were strongly influenced by group size, with non-solitary individuals spending more time close and exhibiting more exploration overall. Through social observations and learning, coyotes in larger groups can learn that a perceived risky interaction may be safe. For example, in a captive study, coyotes that watched a demonstrator solve a puzzle box were found to be less neophobic by spending less time approaching the object, as well as more persistent working on the puzzle box^91^. Within an urban landscape, this reduction in perceived risk can be further mediated by sentinel behavior (or synchronized vigilance), where one individual is vigilant while the other engages in a risky activity^92–94^, such as foraging in an open landscape. Yet, we did not detect an effect of group size on vigilance in our analysis, nor when we examined the effect of group size on max time spent alert post-analysis. Though a more structured design is needed to test for sentinel behavior in urban coyotes, our results suggest that conspecific presence (e.g., risk dilution through observation) is more influential on individual risk-taking than sentinel behavior in pairs or larger groups.

Surprisingly, we did not detect an effect of environmental health on coyote risk-taking. Though current work in birds^37,38^ and fish^39^ demonstrates that individuals who face greater exposure to pollutants have altered risk-taking, the relationship between pollution and risk-taking is far from consistent^95–97^. For example, although urban great tits (*Parus major*) were slower explorers at sites with more metal pollution, aggression and nest defense showed no relationship to metal pollution^37^. Similarly, great tit neophobia was unrelated to toxic metal exposure but was more related to urban disturbances^95^. We may not have detected an effect of pollution on coyote behavior due to the coarseness of our environmental hazard variables at the census tract level, which may not adequately capture the intensity of exposure coyotes face to pollutants at the site-level. Coyotes may be exposed to, for example, air pollutants that vary at very fine spatial scales, such as PM_2.5_^98,99^_,_ in their local habitat. Hence, to disentangle the effect of environmental contamination on coyote risk-taking within cities, a better approach may be to sample locally via air quality monitors and soil samples alongside blood samples to understand site-level exposure to pollutants and how they may be related to coyote behavior.

Our findings point to several potential mechanisms to infer why coyote risk-taking varies as a function of human density. First, habituation or learning may be driving elevated risk-taking in areas of high human density. Through repeated exposure of novel stimuli (e.g., physical, visual), coyote responses to risky stimuli (e.g., novel objects, loud noises) may diminish, leading to no marked behavioral differences between the absence and presence of a novel object. Second, these findings point to differences in the perception of overall risk between low and high human density areas. Areas of high human density have reduced predator assemblages, which can promote boldness and exploration^19,20^, and coyotes in high human density areas encounter risk more frequently than other conspecifics, including vehicles, people, and other disturbances associated with cities^100,101^. Hence, because coyotes must mitigate risk frequently, they are able to assess and determine the threat of novelty and adjust behaviorally if necessary. In our study, this is reflected by coyotes in high human density areas not adjusting their behaviors in the face of novelty, unlike their counterparts in areas with lower human densities who reduced their risk-taking when presented with novelty via reductions in exploration and time spent close. Lastly, development is also a salient factor that could be driving our behavioral observations. Captive research has shown that bold coyotes produce litters that are even bolder^84^ and thus, coyote pups that are raised by bold parents likely have personality traits that yield riskier behaviors. Regardless of the mechanism, our results suggest that behavioral strategies in coyotes differ across urban landscapes as a function of human density, rather than being monotonic.

As an urban apex predator, differences in coyote activity and risk-taking hold implications for community ecology, fitness, and human-carnivore interactions. First, lower coyote detections in areas with high human density and high pollution may lead to bolder and more exploratory phenotypes in subordinate carnivores, such as raccoons (*Procyon lotor*) and foxes (*Vulpes ssp.*). Additionally, a decrease in coyote presence could lead to higher densities of rodents in areas with poorer environmental health and higher human densities, which could lead to spillover of zoonotic diseases or potential infestation in homes with poor infrastructure. Future research should explore how coyote presence, in addition to landscape variables such as human density, influences mesocarnivore risk-taking within urban environments as well as pest prevalence. Second, elevated coyote risk-taking in high human density areas may lead to higher fitness, allowing individuals to enter and establish themselves in areas that less risky individuals are unable to access. Moreover, these behavioral shifts allow coyotes to exploit resources that may be difficult to access due to human presence or infrastructure. However, these same behavioral shifts may further enshrine their social niche in society as a species of conflict concern. Thus, implementing policies surrounding factors associated with higher human population density may be ideal, including education on coyote behavior, nonlethal deterrents, reducing loose trash and handfeeding, and encouraging on-leash dogs to promote human-carnivore coexistence.

In summary, we provide evidence to suggest marked differences in coyote behavioral traits within cities. We demonstrated that coyote detections varied as a function of pollution and human densities, while coyote risk-taking varied with differences in human density as well as group size. Further research is needed to disentangle the exact mechanisms that lead to changes in behavioral strategies and whether these strategies are consistent across other urban mesocarnivores. Our results provide critical insight into urban coyote behavioral ecology, creating a foundation to further explore how intra-city variation influences traits that predict individual success in urban areas, and what these behavioral changes mean for human-carnivore interactions in cities.

## Supplementary Information

Supplementary Information.

## Data Availability

The data analyzed for the study are available from the corresponding author on reasonable request.
