# Supplementary material for: Differential effects of human density, environmental health, and group size on urban coyote detection, boldness, and exploration
Source: Sci Rep. 2025 Oct 30;15:38072. doi: 10.1038/s41598-025-21946-y (PMC12575792; doi:10.1038/s41598-025-21946-y)
Supplement: Supplementary file 1 — Supplementary Information. [file 41598_2025_21946_MOESM1_ESM.docx]

# **Supplemental Information for**

Differential effects of human density, environmental health, and group size on urban coyote detections and risk-taking

Cesar O. Estien, Lauren A. Stanton, Christopher J. Schell

**Contents:**

Pages S1-12

Figures S1-6

Table S1-2

# **Methods**

*Data Cleaning*

Once behavioral data was extracted, we manipulated our data to track instances where behaviors occurred but were not coded by the observer due to limitations in our coding software. For example, an individual may not be vigilant during an observation and thus was vigilant for 0 seconds. However, this information is not captured by BORIS. Thus, we inserted these instances where behavior that was not observed into our data frame to capture the absence of the behaviors of interest.

# **Figures and Tables**

Figure S1. The distribution of pollution burden (triangle, red) and population density (circle, blue) metrics (x-axis) in our study area. Pollution burden is a percentile and scaled between 0-100. For visualization purposes, human population density is also shown as a percentile.

Figure S2. Correlation matrix between variables considered. All variables were extracted within a 500-meter buffer.

Figure S3. Correlation between the number a days a camera was active at a site and the number of coyote detections.


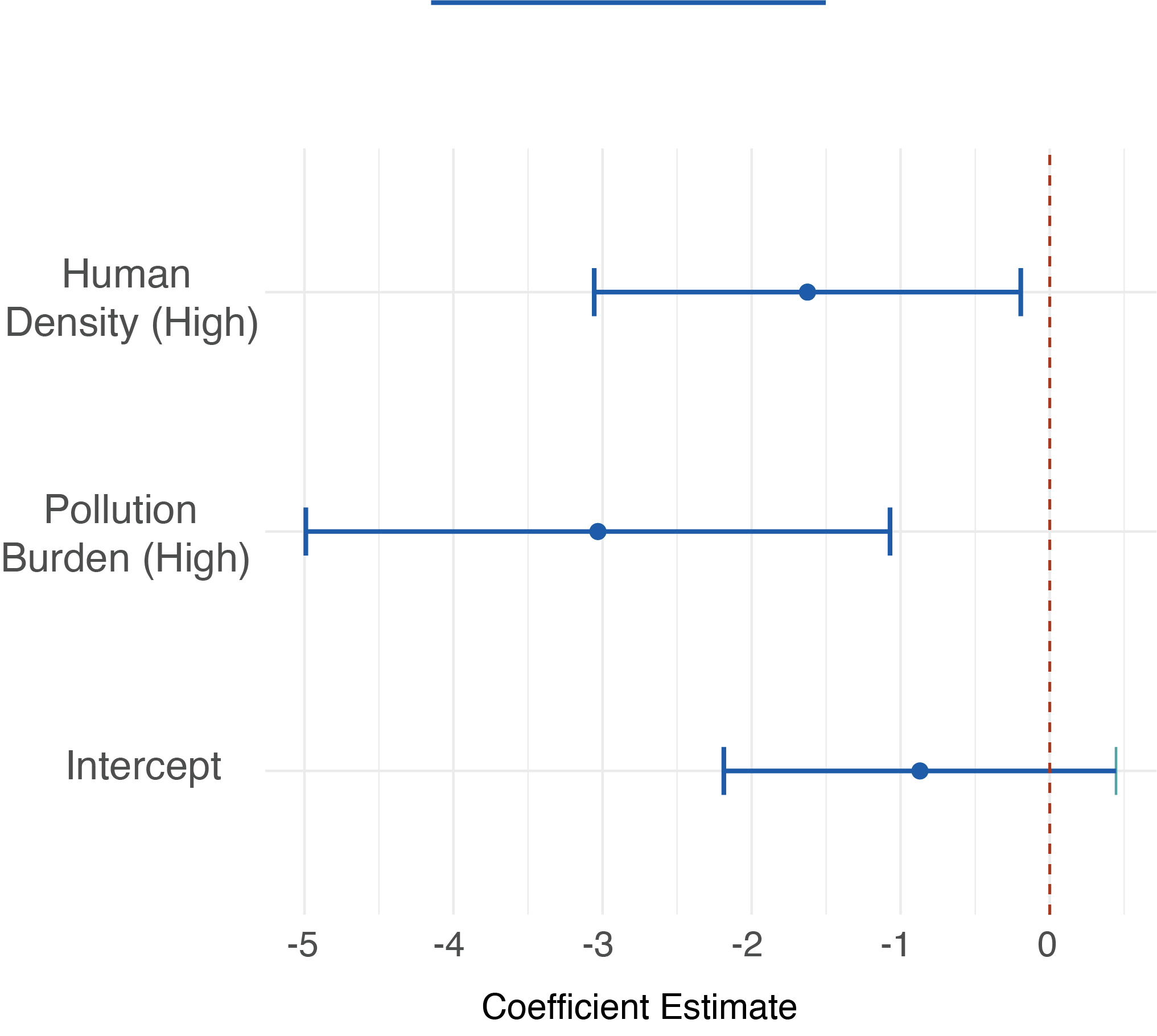


Figure S4. Coefficient estimates for fixed effects in our coyote detection models. Points represent the estimate and whiskers are 95% confidence intervals. Factors that cross the center dashed line are non-significant factors.

Figure S5. Box plot showing coyote detections across human density categories (left panel) and pollution burden categories (right panel). Purple boxes represent the lower category for each variable while blue boxes represent the higher category for each variable. Black diamonds represent the mean.

Figure S6. Correlation plot showing the relationship between the three behaviors of interest: time spent alert, time spent close, and total exploration. Deeper blue colors represent strong, positive correlations while deeper red colors represent strong, negative correlations.

Table S1. Ethogram.

| **Behavior** | **Description** |
| --- | --- |
| Alert | The focal species is alert by exhibiting one or multiple behaviors. For coyotes, individuals may flinch, have its tail tucked, have its ears perked, move hesitantly and/or be crouched while approaching the attractant/object. |
| Close | The focal species is within one body length of the novel object, including physical position directly on the attractant or within the object. |
| Far | The focal species is NOT within one body length of the novel object |
| Touch | The focal species physically touches the object. |
| Out of sight | The focal species is no longer within the video frame (i.e., not visible). |
| Dig | The focal species extends its front leg forward and then drags its paw across the ground toward its body. This occurs within one body length of the object. A separate dig event is considered each time an animal extends its appendage or switches appendages (left paw to right paw, both paws to one paw). |
| Other | The focal species exhibits a behavior not defined above |
| Sniff | The focal species is sniffing within one body length of the object, and its nose is pointed towards the object/ground. |
| Through | The focal species passes through the object |

Table S2. Coyote risk-taking model selection results. K is the number of parameters for each model, ΔAICc is the difference in AICc score between the best model and the model being compared, Weight (0-1) is the AICc weight of evidence in favor of a given model, and LL is the log-likelihood of each model. The best-performing model is listed first per behavior.

| **Behavior** | **Model** | **K** | **ΔAICc** | **Weight** | **LL** |
| --- | --- | --- | --- | --- | --- |
| **Time spent alert** | Human Density * Treatment | 8 | 0.00 | 0.72 | -563.96 |
|  | Global | 11 | 3.88 | 0.10 | -562.68 |
|  | Human Density | 6 | 5.07 | 0.06 | -568.61 |
|  | Treatment | 6 | 5.78 | 0.04 | -568.97 |
|  | Human Density + Pollution Burden | 7 | 6.29 | 0.03 | -568.17 |
|  | Human Density + Pollution Burden + Group Size | 8 | 7.31 | 0.02 | -567.62 |
|  | Group Size + Treatment | 7 | 7.46 | 0.02 | -568.75 |
|  | Pollution Burden * Treatment | 8 | 8.72 | 0.01 | -568.32 |
|  | Null (Observation Number Only) | 5 | 15.56 | 0.00 | -574.90 |
|  | Pollution Burden | 6 | 16.24 | 0.00 | -574.20 |
|  | Group Size | 6 | 17.55 | 0.00 | -574.85 |
| **Time spent close** | Global | 11 | 0.00 | 0.87 | -765.15 |
|  | Human Density * Treatment | 8 | 3.97 | 0.12 | -770.36 |
|  | Group Size + Treatment | 7 | 8.66 | 0.01 | -773.77 |
|  | Treatment | 6 | 14.76 | 0.00 | -777.87 |
|  | Pollution Burden * Treatment | 8 | 14.95 | 0.00 | -775.85 |
|  | Human Density + Pollution Burden + Group Size | 8 | 39.98 | 0.00 | -788.36 |
|  | Human Density + Pollution Burden | 7 | 50.56 | 0.00 | -794.71 |
|  | Human Density | 6 | 51.17 | 0.00 | -796.08 |
|  | Group Size | 6 | 57.36 | 0.00 | -799.17 |
|  | Null (Observation Number Only) | 5 | 67.08 | 0.00 | -805.07 |
|  | Pollution Burden | 6 | 67.97 | 0.00 | -804.47 |
| **Total Exploration** | Global | 11 | 0.00 | 0.81 | -304.08 |
|  | Group Size + Treatment | 7 | 3.63 | 0.13 | -310.18 |
|  | Human Density * Treatment | 8 | 5.65 | 0.05 | -310.13 |
|  | Treatment | 6 | 10.28 | 0.00 | -314.55 |
|  | Pollution Burden * Treatment | 8 | 13.10 | 0.00 | -313.86 |
|  | Human Density + Pollution Burden + Group Size | 8 | 46.69 | 0.00 | -330.65 |
|  | Group Size | 6 | 53.36 | 0.00 | -336.10 |
|  | Human Density | 6 | 56.00 | 0.00 | -337.42 |
|  | Human Density + Pollution Burden | 7 | 57.78 | 0.00 | -337.25 |
|  | Null (Observation Number Only) | 5 | 62.98 | 0.00 | -341.95 |
|  | Pollution Burden | 6 | 64.78 | 0.00 | -341.81 |

Table S3. Coyote detection model results. 

| **Model** | R^2^_c_ | Term | Estimate | SE | Pr (>\|z\|) | 95% CI |
| --- | --- | --- | --- | --- | --- | --- |
| **Coyote Detections** | 0.391 | Intercept | -0.871 | 0.671 | 0.195 | -2.186, 0.445 |
|  |  | Human Density (High) | -1.625 | 0.7301 | < 0.05 | -3.056, -0.194 |
|  |  | Pollution Burden (High) | -3.031 | 1.000 | < 0.01 | -4.991, -1.071 |
